# Gender and the Digital Divide Across Urban Slums of New Delhi, India: Cross-Sectional Study

**DOI:** 10.2196/14714

**Published:** 2020-06-22

**Authors:** Ashish Joshi, Bhavya Malhotra, Chioma Amadi, Menka Loomba, Archa Misra, Shruti Sharma, Arushi Arora, Jaya Amatya

**Affiliations:** 1 Graduate School of Public Health and Health Policy City University of New York New York, NY United States; 2 Foundation of Health care Technologies Society Delhi India

**Keywords:** gender, digital divide, mobile phone, internet access, text messaging, slums

## Abstract

**Background:**

Disparities in access to specific technologies within gender groups have not been investigated. Slum settings provide an ideal population to investigate the contributing factors to these disparities.

**Objective:**

This study aimed to examine gender differences in mobile phone ownership, internet access, and knowledge of SMS text messaging among males and females living in urban slum settings.

**Methods:**

A convenience sampling approach was used in sample selection from 675 unnotified slums. A total of 38 slum sites were then selected across four geographic zones. Of these, 10% of the households in each slum site was selected from each zone. One household member was interviewed based on their availability and fulfillment of the eligibility criteria. Eligible individuals included those aged 18 years and above, residing in these slums, and who provided voluntary consent to participate in the study. Individuals with mental or physical challenges were excluded from the study.

**Results:**

Our results showed that females were half as likely to own mobile phones compared with males (odds ratio [OR] 0.53, 95% CI 0.37-0.76), less likely to have internet access (OR 0.79, 95% CI 0.56-1.11), or know how to send text messages (OR 0.93, 95% CI 0.66-1.31). The predictors of mobile phone ownership, internet access, and text messaging *between* males and females included age, individual education, housing type, and the number of earning members in a household in the adjusted analysis. Among males, the number of earning members was a predictor of both mobile phone ownership and text messaging, whereas household education was a predictor of both internet access and text messaging. Age and individual education only predicted internet access, whereas housing type only predicted text messaging. Among females, household education was a predictor of all the technology outcomes. Age and type of toilet facility only predicted mobile phone ownership; housing type only predicted internet access whereas television ownership with satellite service and smoking behavior only predicted text messaging.

**Conclusions:**

Our study findings showing disparate access to technology *within* gender groups lend support for further research to examine the causal mechanisms promoting these differences to proffer significant solutions. Specifically, our study findings suggest that improving household education is crucial to address the disparate access and usage of mobile phones, the internet, and text messaging among women in slum settings. This suggestion is due to the consistency in household educational level as a predictor across all these technology indicators. In addition, the mechanisms by which the number of household earning members influences the disparate access to technology among men call for further exploration.

## Introduction

### Digital Divide

Information and Communication Technology (ICT) plays a major role in fostering access to knowledge and key services across various sectors. ICT proliferation has been associated with increasing economic benefits, including new economic opportunities, increased trade, higher productivity, and lower costs. ICT is in a continuous state of advancement, evolution, and rapid diffusion at record-setting rates. For instance, global internet penetration rose from around 6% to almost 50% between 2000 and 2016, with penetration rates exceeding 90% in developed countries [1]. The term *digital divide* was coined in the 1990s by Lloyd Morriset (President of the Markle Foundation) to describe these inequalities, which depict a divide between the *information-haves and have-nots* [2]. The digital divide connotes disparate access to information across individuals with and without access to the internet, and more broadly, ICT as well as the general media [2]. However, the digital divide is most commonly used for indicating the availability of internet access at an affordable cost and quality [3,4]. Disparate patterns in technology access are frequently measured by internet access, penetration, the number of internet users, household ownership of computers, and mobile phone usage [5].

### Background

The factors impacting the digital divide were conceptualized using the resources and appropriation theory developed by Van Dijk [5]. This theoretical framework depicts a causal model of the interplay between 1) individual and societal inequalities, 2) distribution of resources, 3) access to ICTs, and 4) societal participation. Specifically, the differential growth in ICT access and usage across countries in varied settings has been attributed to gross inequalities at the individual and societal levels. These are observed across age distributions, gender, race or ethnicity, income, literacy, personality, health, household conditions, and socioeconomic status (SES) [5]. The inequalities subsequently produce an unequal distribution of resources that promote unequal access to technology. Unequal access to technology is also dependent on the type of technology (ie, basic phone with limited functionality such as the absence of cameras, smartphone, computer, and other advanced systems). Unequal access to technology contributes to unequal societal participation, which, in turn, reinforces the existing inequalities [5]. Such inequalities tend to be more pronounced in marginalized settings, notably in urban slum settings in developing countries, which constitute a hub of economic disadvantage.

Although a variety of individual-level determinants of ICT inequalities exist, gender remains one that is of primary importance, as reflected in the United Nations (UN) Sustainable Development Goal 5: Gender Equality. The Sustainable Development Goal 5B target is to enhance the use of enabling technology, in particular ICT technology, to promote the empowerment of women [6]. Unequal access to technology among men and women constitutes one of the most striking aspects of the digital divide. The impact of the digital divide on gender has been widely studied in various developing and developed economies [7]. According to the International Telecommunication Union (ITU), on average, women are 16 percentage points less likely to use the internet compared with their male counterparts. This gender-gap is consistent globally, varying between 11% and 19% in Nigeria, Tanzania, India, Pakistan, and Japan, with differences as high as 31% in the least developed settings [7]. Gender gaps in these settings have been attributed to a variety of determinants, including disproportionate access to education among young women, which impairs their literacy levels. Further determinants include limited institutional opportunities for ICTs, personal safety issues with access to ICTs, and the *leaky pipe phenomenon,* which describes female preference to advance their family’s welfare over their personal development [6]. These barriers are perpetuated by structural factors such as extreme poverty and highly patriarchal societies, as well as psychological barriers such as limited confidence among women in their capacity to learn ICT skills, and the belief that technology is reserved for their male counterparts [7].

### Objectives

Although prior research has established the existence of a digital divide between gender categories, findings of these studies suggest that variations within individual gender categories may be significantly contributing to the existing digital divide [8]. Slum settings provide a unique population to investigate the contributing factors to the disparity in technology access and usage within gender categories. This is because slums represent a hub of staggering economic disparities, which tend to be more diverse than the nonslum populations. In particular, a UN habitat report indicated that slums suffer from higher disease incidence and mortality, which exceed nonslum populations, and these disparities are rarely reflected in the national statistics, thereby masking the extent of the deprivation in slum settings [9]. Essentially, the state of slums constitutes an indicator of prosperous cities [9]. The objective of this study was to examine gender differences in mobile phone ownership, internet access, and knowledge of SMS among males and females living in urban slum settings.

## Methods

### Overview

**Figure 1:**
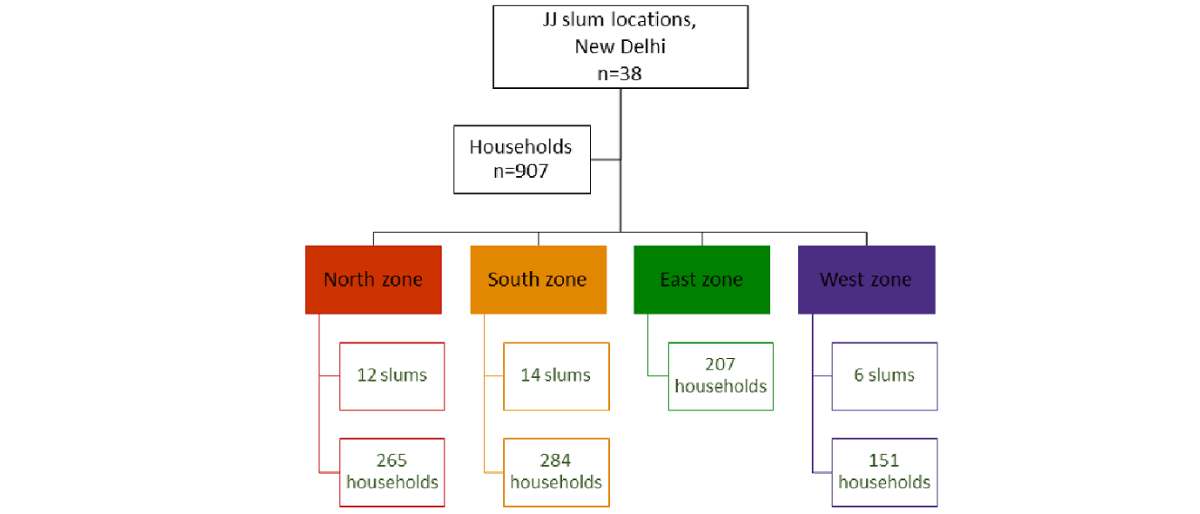
Study participant recruitment.

A cross-sectional study was conducted between June 2016 and January 2017 to assess the impact of the digital divide among women and men residing in urban slums of Delhi, India. The sampling frame used was the *Delhi Urban Shelter Improvement Board Jhuggi-Jhopadi Cluster List of 2015*, which enumerated a total of 675 unnotified urban slums across the four geographic zones (North, South, East, and West) of New Delhi, India. Unnotified slums are slum settlements that are not federally recognized and do not benefit from government subsidies and interventions across slums [10]. A convenience sampling approach was used in identifying 675 unnotified slums, and selecting 38 slum sites across four zones (north zone, n=12; south zone, n=14; east zone, n=6; and west zone, n=6; Figure 1). From each zone, 10% of the households were selected based on proximity to the researcher, ease of access to the slums, and the presence of local collaborators in these slums who could help in navigating them. Of these, 1 member from each household was selected and interviewed based on availability for the interview and fulfillment of the eligibility criteria. Eligible individuals included those who were aged 18 years and above, resident in these slums, and provided voluntary consent to participate in the study. Individuals who did not provide consent and had any mental challenges were excluded from the study. This resulted in a total sample of 907 study participants across all the slums. The Institutional Review Board (IRB) of the Foundation of Healthcare Technologies Society, New Delhi, India, approved the study protocol (IRB number: FHTS/041/2016).

### Variables Assessed

The following variables were assessed:

- Sociodemographic characteristics: age, gender, education, household education, type of family, earning members in the household, housing type, type of toilet facility, television ownership, and healthy behaviors, including smoking and alcohol consumption.
- Living index: information was collected about housing type (concrete, semiconcrete, or not concrete), access to toilet facility (in-house, public place, or open defecation), television ownership, and use of satellite television service.
- High-risk behaviors: information was collected on the reporting of high-risk behaviors, including smoking and alcohol consumption.
- Mobile ownership, internet access, and knowledge of SMS: information was collected about individual or household mobile phone ownership, internet service (mobile phone), and knowledge of SMS text messaging.

### Statistical Analysis

Descriptive analysis was conducted to report means with SDs and frequencies for all continuous and categorical variables, respectively. Association was performed between sociodemographic characteristics, living index, and health behaviors and technology outcomes, including mobile phone ownership, access to the internet, and knowledge of text messaging. Stratified analysis by gender was performed to determine the between sociodemographic characteristics, living index, and health behaviors and technology outcomes, including mobile phone ownership, access to the internet, and knowledge of text messaging. Variables having significant relationship were included in multivariable logistic regression. Multivariate analyses, stratified by gender, were performed to examine variables that were associated with mobile phone ownership, internet access, and knowledge of SMS after adjusting for potential confounders including sociodemographics, healthy behaviors, and living index conditions. The analyses were performed using SAS, AS, Version 9.4 (SAS Software Limited).

## Results

### Study Participant Characteristics

The average age of the study participants was 36 years (SD 13). Almost half of them were between the ages of 18 and 30 years (398/904, 44.0%). More than half of them were females (599/904, 66.3%), and 46.2% (418/904) had not completed high school. Half of them lived in households where the highest level of education was less than a high school diploma (453/904, 50.1%). More than half of the study participants lived in nuclear families (578/904, 64%) and had 1 earning member per household (534/904, 59.1%).

More than half of the study participants resided in houses that had concrete finishing (496/904, 54.9%). Of which, 45.2% (409/904) of the study participants utilized public toilet facilities as their primary source of sanitation. More than two-thirds of the study participants owned a television set (705/904, 77.9%) and had satellite television service (592/904, 65.5%). Then, 22.0% (199/904) of the participants reported smoking and 11.6% (105/904) reported alcohol consumption. The characteristics of the study participants have been previously published [11]. Less than 1% of responses were missing across the technology outcomes assessed (34/85000, 0.04%).

### Mobile Phone Ownership, Internet Service, and Text Messaging Among Study Participants

**Figure 2:**
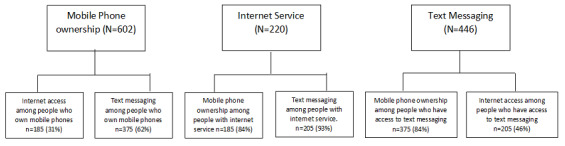
Phone ownership, internet service, and text messaging among the study participants (N=904).

More than half of the study participants owned a mobile phone (602/904, 66.5%). Only 24.3% (220/904) of the study participants had internet service on their mobile phones. Less than half of the study participants had knowledge of sending text messages (446/904, 49.3%; Figure 2).

More than half of the study participants who owned mobile phones had knowledge of sending text messages (375/602, 62.2%). However, less than half of them had internet service on their mobile phones (185/602, 30.7%; Figure 2 and Multimedia Appendix 1). More than two-thirds of the study participants who had internet service owned a mobile phone (185/220, 84.1%), and 93.2% (205/220) of them had knowledge of sending text messages. In all, 84.1% (375/446) of the study participants who knew how to send text messages owned a mobile phone, and less than half of them had access to the internet (205/446, 45.9%; Figure 2).

### Mobile Phone Ownership and Technology Access and Familiarity

#### Variables Associated With Mobile Phone Ownership, Internet Access, and Knowledge of Text Messaging Among the Study Participants

##### Mobile Phone Ownership

More than half of the study participants owned a mobile phone (602/904, 66.3%). A total of 61.7% (365/904) of them were females, 43.8% (264/602) of them were between 18 and 30 years old, and 54.3% (327/602) had not obtained a high school diploma (Multimedia Appendix 1). More than half of them lived in nuclear families having 1 earning member (351/602, 58.3%), and with a household educational level of less than high school (312/602, 51.8%). College education at the individual and household levels were significantly higher among study participants who owned a mobile phone compared with those that did not (*P*<.001).

A total of 61.7% (372/602) of the study participants that owned a mobile phone resided in households with concrete finishing compared with 41.1% (124/302) of those who did not own a mobile phone (*P*<.001). Less than 10% of the participants that owned a mobile phone practiced open defecation (40/602, 6.6%) compared with 25.8% (78/302) of those that did not own a mobile phone (*P*<.001). The majority of mobile phone owners also owned a television (515/602, 85.5%) and had access to a satellite television service (437/602, 72.5%). Smoking (*P*=.50) and alcohol consumption (*P*=.40) were not significantly associated with those owning a mobile phone (Multimedia Appendix 1). Age of the study participant, gender, individual and household education, type of family, number of earning members, housing type, type of toilet facility, and television ownership with satellite service were significantly associated with mobile phone ownership.

##### Internet Access

Less than one-third of the study participants had internet service on their mobile phones or had a household member with internet service (220/904, 24.3%). percent of these, 51.4% (113/220) of those with internet service on their phones were between the ages of 18 and 30 years (*P*=.02; Multimedia Appendix 1). More than half of them were females (127/220, 57.7%) and had not completed high school (116/220, 52.7%). In all, 52.7% (116/220) of them lived in nuclear family settings and had 1 earning family member (110/220, 50.0%). More than two-thirds of them resided in houses with concrete finishing (153/220, 69.5%), and 50.0% (111/220) of them utilized in-house toilet facilities. Then, 78.6% (173/220) of them owned a television with satellite television service (*P*<.001)

The study participants age (*P*=.02), gender (*P*=.002), individual and household education, type of family, number of earning members, housing type (*P*=.02), type of toilet facility, and television ownership with satellite service were significantly associated with internet access on a mobile phone (*P*<.001). Smoking (*P*=.50) and alcohol consumption (*P*=.40) were not significantly associated with having internet service on their mobile phones.

##### Text Messaging

Less than half of the study participants knew how to send text messages (446/904, 49.3%; Multimedia Appendix 1). More than half of them were females (276/446, 61.9%) and had not completed high school (252/446, 56.5%). Almost one-third of the study participants who were knowledgeable about texting had obtained some college education (123/446, 27.5%) compared with 7.0% (31/446) of those who did not know how to send text messages. The majority of them had 1 to 2 earning members in the household. More than two-thirds of them resided in houses with concrete finishing (299/446, 67.0%). Less than half of them utilized public places (205/446, 45.9%) or in-house toilet facilities (212/446, 47.5%) as their primary mode of sanitation. Of these, 6.5% (29/446) practiced open defecation. The majority of them owned a television (402/446, 90.1%) and had satellite television service (347/446, 77.8%). Smoking and alcohol consumption were not significantly associated with the knowledge of text messaging. Age of the study participant, gender, individual and household education, type of family, number of earning members, housing type, type of toilet facility, television ownership with satellite service, and technology usage were significantly associated with the knowledge of text messaging.

#### Predictors of Mobile Phone Ownership, Internet Access, and Text Messaging Among the Study Participants

##### Predictors of Mobile Phone Ownership

Age, household educational level, number of earning members, housing type, and type of toilet facility remained significantly associated with mobile phone ownership (Multimedia Appendix 2). Females had lower odds of mobile phone ownership compared with males (odds ratio [OR] 0.51, 95% CI 0.35-0.75). Study participants in the age group of 41-50 years had the highest odds of mobile phone ownership as compared with those in the 50+ age (OR 1.99, 95% CI 1.07-3.69). Individuals living in households where the highest educational level was less than a high school diploma was associated with lower odds of mobile phone ownership (OR 0.51, 95% CI 0.28-0.94). Similarly, no earning member in a household was associated with lower odds of mobile phone ownership (OR 0.27, 95% CI 0.08-0.91). Living in houses made of concrete was associated with higher odds of mobile phone ownership (OR 1.99, 95% CI 1.13-3.49). In addition, living in households that utilized public places as their primary mode of sanitation was associated with a higher odds of mobile phone ownership (OR 1.56, 95% CI 1.07-2.25), whereas open defecation was associated with a lower odds of mobile phone ownership (OR 0.49, 95% CI 0.29-0.84).

##### Predictors of Internet Access

Age and household education remained significantly associated with internet access (Multimedia Appendix 2). Females had lower odds of internet access compared with males (OR 0.65, 95% CI 0.44-0.96). Study participants aged between 18 and 30 years had the highest odds of internet access across age groups (OR 2.19, 95% CI 1.15-4.19). Households in which the highest level of education attained was high school were associated with a lower odds of internet access (OR 0.49, 95% CI 0.28-0.87).

##### Predictors of Text Messaging

Age, individual education, household education, number of earning members in the household, housing type, and satellite television service remained significantly associated with text messaging (Multimedia Appendix 2). Study participants between the ages of 41 and 50 had the highest odds of text messaging (OR 2.12, 95% CI 1.13-3.97). Households in which no member had obtained any schooling was associated with lower odds of text messaging (0.22, 95% CI 0.05-0.93). Living in houses made of concrete (OR 2.05, 95% CI 1.13-3.72) and having a satellite television service were associated with higher odds of text messaging (OR 1.94, 95% CI 1.12-3.33).

### Stratified Analysis of Mobile Phone Ownership, Internet Access, and Text Messaging Across Gender Categories (Bivariable Analysis)

#### Mobile Phone Ownership Stratified by Gender

A total of 39.4% (237/602) of males and 60.1% (365/602) of females owned mobile phones. Gender differences in mobile phone ownership were significant across age groups (*P*=.002), educational status of the participant (*P*<.001), housing type (*P*=.02), smoking (*P*=.001), and alcohol consumption (*P*=.02; Multimedia Appendix 3). Of which, 60.1% (365/602) of the females owned mobile phones compared with 39.3% (237/602) males; 8.4% (20/237) of the males who owned a mobile phone had obtained some college education compared with 5.2% (19/365) of the females (*P*<.001); 8.5% (31/365) of the females who owned a mobile phone resided in houses with nonconcrete finishing compared with 5.1% (12/237) of the males (*P*=.02). One-third of the males who owned a mobile phone reported smoking (n=73), compared with 17.3% (63/365) of the females. And 16.9% (40/237) of the males reported alcohol consumption compared with 10.4% (38/365) of the females. Gender differences were not significant by household education (*P*=.74), family type (*P*=.97), total earning members (*P*=.19), type of toilet facility (*P*=.91), and television ownership (*P*=.29)

#### Access to Internet Services Stratified by Gender

A total of 42% (93/220) of the males had access to internet services as compared with 57.7% (127/220) of females. Gender differences in internet access were significant across educational levels (*P*<.001) and housing type (*P*=.01; Multimedia Appendix 4). Almost one-quarter of the males had obtained some college education (21/93, 23%) compared with 9% (11/127) of females (*P*<.001) More than two-thirds of the females (94/127, 74%) resided in houses made of concrete compared with 64% (59/93) of the males. Gender differences in access to the internet were not significant by age (*P*=.09), household education (*P*=.25), family type (*P*=.78), toilet facility (*P*=.73), television ownership (*P*=.09), smoking (*P*=.05), and alcohol consumption (*P*=.92) .

#### Text Messaging Stratified by Gender

A total of 38.1% (170/446) of males were familiar with sending text messaging as compared with 61.8% (276/446) of females. Gender differences were significant across educational groups (*P*<.001), total earning members in the household (*P*=.03), housing type (*P*<.001), smoking behaviors (*P*<.001), and alcohol consumption (*P*<.04; Multimedia Appendix 5). Almost half of the study participants capable of texting were aged 18 to 30 years. Of which 47.1% (130/276) of them were females compared with 45.9% (78/170) of them that were males; 14.2% (24/170) of the males were 50 years or older compared with 7.9% (22/276; *P*=.03) of females; and 12.3% (21/170) of the males had obtained some college education compared with 7.6% (21/276) of the females (*P*<.001). Less than half of the males had 1 earning member (83/170, 48.8%) compared with 58.7% (162/276) of the females. More than two-thirds of the females (201/276, 72.8%) resided in houses made of concrete compared with 57.6% (98/170) of the males, 30.6% (52/170) of the males reported smoking (*P*<.001), and 15.9% (27/170) of the males reported drinking (*P*=.04).

### Factors Associated With Mobile Phone Ownership, Internet Access, and Text Messaging Stratified by Gender (Multivariable Analysis)

Age (*P*=.002), educational status (*P*=.003), and type of housing (*P*=.004) remained predictors of mobile phone ownership between males and females (Multimedia Appendix 6). Being older than 30 years (OR 2.19, 95% CI 1.35-3.58) and living in houses made of concrete (OR 2.49, 95% CI 1.50-4.14) were also significant predictors of mobile phone ownership. Educational status was the only significant predictor of internet access between males and females (*P*<.001). Older age (*P*=.004), less than high school education (*P*<.001), 1 household earning member, or less (*P*=.02) were associated with a lesser odds of text messaging between males and females. Living in houses made of concrete was associated with greater odds of text messaging (OR 2.69, 95% CI 1.55-4.69) (Multimedia Appendix 6).

### Within-Gender Variation Related to Mobile Phone Ownership, Internet Access, and Text Messaging

#### Within-Gender Variation Related to Mobile Phone Ownership

Educational status of the individual (*P*<.001), household educational attainment (*P*<.001), total earning members in the household (*P*<.001), housing type (*P*<.001), type of toilet facility (*P*<.001), television ownership (*P*<.001), having a satellite television service (*P*<.001), and smoking (*P*=.02) were significantly associated with mobile phone ownership among males (Multimedia Appendix 7). More than half of the males who owned mobile phones had not obtained a high school education (154/237, 64.9%); resided in households where the highest level of education was less than high school (127/237, 53.6%), and had 1 earning member (129/237, 54.4%). More than half of them resided in houses with concrete finishing (137/237, 57.8%), and half of them utilized public places as the main source of sanitation (n=119). More than two-thirds of them owned a television and had satellite television service. One-third of the male participants who owned a mobile phone were smokers (n=73/237). Age (*P*=.30), type of family (*P*=.31), and alcohol consumption (*P*=.89) were not significantly associated with mobile phone ownership among men (Multimedia Appendix 7).

Age of the study participants, individual and household educational attainment, type of family, housing type, type of toilet facility, television ownership, and having a satellite television service were significantly associated with mobile phone ownership among female study participants (*P*<.001; Multimedia Appendix 6). Almost half of the females who owned a mobile phone were between 18 and 30 years old (167/365, 45.2%) and had not obtained a high school diploma (173/365, 47.4%; *P*<.001). More than half of them resided in households where the highest level of education attained was less than a high school diploma (185/365, 50.7%), and 64% (234/365) of them lived in nuclear families and in houses with concrete finishing (n=235). Less than half of them utilized public places as their main source of sanitation (177/365, 48.5%); 84.9% (310/365) of them owned a television, and 70.1% (256/365) of them had satellite television service. The number of earning members in the household (*P*=.52), smoking (*P*=.24), and alcohol consumption (*P*=.14) were not significantly associated with mobile phone ownership among women (Multimedia Appendix 7).

#### Within-Gender Variation Related to Internet Access

Age of the study participant (*P*=.002), educational level (*P*<.001), household education (*P*<.001), total earning members in the household (*P*=.003), and housing type (*P*=.01) were significantly associated with mobile phone internet access among men (Multimedia Appendix 8). More than half of the males who had internet access on their mobile phones were aged 18 to 30 years (*P*=.002). Almost a quarter of them had obtained some college education (21/93, 23%). Almost half of them lived in households where the highest level of education attained was college (40/93, 43%), and 41% (38/93) of them had 1 earning member in the household.

The educational level of the study participant, household education, type of family, housing type, type of toilet facility, television ownership, and satellite television service were significantly associated with mobile phone internet access among the females (*P*<.05). Of these, 9.0% (11/122) of females who had internet access had obtained some college education (*P*<.001). More than one-third of the females with internet access lived in households where a college degree had been attained (45/127, 35.4%; *P*<.001). Half of them came from nuclear families (64/127, 50.4%). More than two-thirds resided in houses made of concrete (94/127, 74.0%) and primarily used in-house toilet facilities (67/127, 52.8%). Almost all of them owned a television (117/127, 92.1%) and had satellite television service (103/127, 81.1%). Age (*P*=.22), total earning members in the household (*P*=.33), smoking (*P*=.29), and alcohol consumption (*P*=.05) were not significantly associated with internet access among females (Multimedia Appendix 8).

#### Within-Gender Variation Related to Text Messaging

The study participant’s education (*P*<.001), household education (*P*<.001), total earning members (*P*=.001), housing type (*P*<.001), toilet facility (*P*<.001), television ownership (*P*<.001), and satellite television service (*P*<.001) were significantly associated with the knowledge of text messaging among males (Multimedia Appendix 9). More than half of the males who were familiar with text messages had not completed high school (112/170, 65.9%; *P*<.001). Half of them lived in households where the highest level of education attained was less than a high school diploma (85/170, 50.0%; *P*<.001). Less than half of them had 1 earning member (83/170, 48.8%), 57.6% (98/170) of them lived in houses made of concrete, and 50.0% (85/170) of them utilized public places as their primary source of sanitation. The majority of them owned a television (153/170, 90.0%) and had satellite television service (134/170, 78.8%; *P*<.001). High-risk behaviors, including smoking (*P*=.15) and alcohol consumption (*P*=.66) were not significantly associated with text messaging among males.

Age of the study participant (*P*=.02), education (*P*<.001), household education (*P*<.001), type of family (*P*=.002), housing type (*P*<.001), toilet facility (*P*<.001), television ownership (*P*<.001), satellite television service (*P*<.001), and smoking (*P*=.03) were significantly associated with the knowledge of text messaging among females (Multimedia Appendix 9). Almost half of the females who were familiar with sending text messages were between the ages of 18 and 30 years (130/276, 47.1%; *P*=.02). More than half of them had not completed high school (140/276, 50.7%), and less than half of them resided in households where the highest level of education was less than a high school diploma (121/276, 43.8%; *P*<.001). More than half of them resided in nuclear families (158/276, 57.2%) with 1 earning member (162/276, 59%). Half of them lived in houses with in-house toilet facilities; 72.8% (201/276) of the females who were familiar with sending text messaging resided in houses made of concrete compared with 43.0% (139/323) who were not capable of texting (*P*<.001). The majority of them owned a television (249/276, 90.2%) and had satellite television service (213/276, 77.1%); 12.3% (34/276) of females who were capable of texting reported smoking behaviors compared with 18.9% (61/323) who were not capable of texting (*P*=.03).

#### Multivariable Analysis of Factors Associated With Mobile Phone Ownership Within Males and Females

The number of household-earning members and the type of toilet facility in a household remained significantly associated with mobile phone ownership among males (*P*=.01; Table 1). Males who had no earning members in the household had a lesser odds of mobile phone ownership (OR 0.06, 95% CI 0.01-0.54; *P*=.01), and a higher odds of practicing open defecation as their primary source of sanitation (OR 2.88, 95% CI 1.09-7.63; *P*=.03).

Age of the study participant (*P*=.01), household education (*P*=.001), and type of toilet facility (*P*=.03) remained significantly associated with mobile phone ownership among females. Females between the ages of 31 and 40 years had the highest odds of mobile phone ownership among all the age groups (OR 2.418, 95% CI 1.243-4.703; *P*=.009). Females in households where no one had obtained any schooling had the lowest odds of mobile phone ownership (OR 0.27, 95% CI 0.12-0.59). In addition, females living in households that utilized public places as their primary mode of sanitation had a higher odds of mobile phone ownership (OR 1.59, 95% CI 1.04-2.43; *P*=.03), whereas those practicing open defecation had a lower odds of mobile phone ownership (OR 0.47, 95% CI 0.26-0.87).

**Table 1:** Multivariable analysis showing predictors of mobile phone ownership within the male and female study participants (N=904).

Variables		Mobile phone ownership			
		Males (n=305)		Females (n=599)	
		OR (95% CI)	*P* value	OR (95% CI)	*P* value
**Age (years)**					
	18-30	—^a^	—	1.89 (0.9-3.59)	.05
	31-40	—	—	2.42 (1.24-4.70)	.01
	41-50	—	—	2.16 (1.01-4.59)	.05
	50+^b^	—	—	—	—
**Education**					
	No school	0.51 (0.08-3.46)	.49	0.714 (0.213-2.394)	.58
	Incomplete school	2.16 (0.32-14.49)	.45	1.219 (0.369-4.031)	.75
	High school diploma	0.76 (0.09-6.05)	.79	1.34 (0.319-5.619)	.69
	Some college or college graduate^b^	—	—	—	—
**Household education**					
	No school	0.54 (0.11-2.72)	.46	0.27 (0.12-0.59)	.001
	Incomplete school	0.52 (0.13-2.13)	.36	0.54 (0.27-1.06)	.07
	High school diploma	1.29 (0.27-6.14)	.75	0.56 (0.26-1.19)	.13
	Some college or college graduate^b^	—	—	—	—
**Type of family**					
	Broken^b^	—	—	—	—
	Extended	—	—	0.6 (0.10-3.93)	.63
	Joint	—	—	1.39 (0.29-6.78)	.68
	Nuclear	—	—	1.38 (0.29-6.64)	.68
**Total earning members in the household**					
	No earning member	0.06 (0.01-0.54)	.01	—	—
	1 earning member	0.98 (0.32-2.99)	.97	—	—
	2 earning members	1.09 (0.32-3.69)	.89	—	—
	3 or more earning members^b^	—	—	—	—
**Housing type**					
	Nonconcrete^b^	—	—	—	—
	Concrete	0.41 (0.14-1.17)	.09	1.37 (0.71-2.62)	.35
	Semiconcrete	2.05 (0.86-4.90)	.11	0.77 (0.40-1.48)	.43
**Type of toilet facility**					
	In-house^b^	—	—	—	—
	Public place	2.23 (0.72-6.93)	.16	1.59 (1.04-2.43)	.03
	Open defecation	2.88 (1.09-7.63)	.03	0.47 (0.26-0.87)	.02
**Television ownership**					
	No^b^	—	—	—	—
	Yes	1.27 (0.34-4.81)	.72	1.06 (0.54-2.07)	.87
**Television ownership with satellite television service**					
	No^b^	—	—	—	—
	Yes	3.30 (0.93-11.69)	.06	1.28 (0.71-2.31)	.40
**Smoking**					
	No^b^	—	—	—	—
	Yes	0.61 (0.30-1.22)	.16	—	—

^a^Empty cells indicate that the variables were not significant in the bivariate analysis for the respective gender group.

^b^Reference group.

### Multivariable Analysis of Factors Associated With Internet Access Within Males and Females

Age of the study participant (*P*=.04), education (*P*=.01), and household education (*P*<.001) remained significantly associated with mobile phone internet access among males (Table 2). Males between the ages of 18 and 30 years had higher odds of internet access (OR 2.60, 95% CI 1.02-6.67; *P*=.04). The odds of internet access were lower among males with no schooling (OR 0.09, 95% CI 0.02-0.57; *P*=.01) or incomplete schooling (OR 0.16, 95% CI 0.03-0.83; *P*=.03). Living in households where the highest educational level was at most a high school diploma was associated with a lower odds of internet access (OR 0.29, 95% CI 0.09-0.89; *P*=.03).

Household educational level (*P*<.001) and housing type (*P*=.03) remained significantly associated with mobile phone internet access among females. Living in households where the highest educational level was less than a high school diploma was associated with a lower odds of internet access (OR 0.16, 95% CI: 0.09-0.31; *P*<.001). In addition, the odds of internet access were lower among females living in houses made of semiconcrete (OR 0.38, 95% CI 0.16-0.92).

**Table 2:** Multivariable analysis showing predictors of internet access within the male and female study participants (N=220).

Variables			Internet access														
			Males (n=305)									Females (n=599)					
			OR (95% CI)		*P* value								OR (95% CI)		*P* value		
**Age (years)**																	
	18-30	2.60 (1.02-6.67)			.04								—^a^			—	
	31-40	1.57 (0.56-4.41)			.39								—			—	
	41-50	0.86 (0.28-2.69)			.79								—			—	
	50+^b^	—			—								—			—	
**Education**																	
	No school			0.09 (0.02-0.57)		.01								0.65 (0.22-1.93)			.44
	Incomplete school			0.16 (0.03-0.83)		.03								1.82 (0.63-5.28)			.27
	High school diploma			0.34 (0.06-2.02)		.24								1.12 (0.29-4.29)			.86
	Some college or college graduate^b^			—		—								—			—
**Household education**																	
	No school			0.26 (0.05-1.28)		.09								0.16 (0.06-0.42)			<.001
	Incomplete school			0.23 (0.08-0.61)		.004								0.16 (0.09-0.31)			<.001
	High school diploma			0.29 (0.09-0.89)		.03								0.68 (0.34-1.33)			.26
	Some college or college graduate^b^			—		—								—			—
**Type of family**																	
	Broken^b^			—		—				—							—
	Extended			—		—				1.27 (0.11-14.50)							.85
	Joint			—		—				1.98 (0.21-18.69)							.55
	Nuclear			—		—				0.80 (0.09-7.47)							.85
**Total earning members in the household**																	
	No earning member			0.17 (0.01-2.22)		.18								—			—
	1 earning member			0.45 (0.18-1.14)		.09								—			—
	2 earning members			1.29 (0.50-3.34)		.59								—			—
	3 or more earning members^b^			—		—								—			—
**Housing type**																	
	Nonconcrete^b^			—		—			—								—
	Concrete			2.51 (0.61-10.36)		.20			0.79 (0.35-1.79)								.57
	Semiconcrete			2.62 (0.64-10.74)		.18			0.38 (0.16-0.92)								.03
**Type of toilet facility**																	
	In-house^b^			—		—					—						—
	Public place			1.94 (0.90-4.16)		.09					0.70 (0.42-1.17)						.17
	Open defecation			—		—					1.25 (0.53-2.92)						.61
**Television ownership**																	
	No^b^			—		—	—										—
	Yes			—		—	1.94 (0.72-5.23)										.19
**Television ownership with satellite television service**																	
	No^b^			—		—				—							—
	Yes			—		—		1.40 (0.67-2.95)									.37

^a^Empty cells indicate that the variables were not significant in the bivariate analysis for the respective gender group.

^b^Reference group.

### Multivariable Analysis of Factors Associated With Knowledge of SMS Text Messaging Within Males and Females

Household educational level (*P*<.001), number of earning members (*P*=.01), and housing type (*P*=.02) remained significantly associated with the knowledge of text messaging among males (Table 3). Males from households who had no schooling (OR 0.05, 95% CI 0.02-0.18) or less than high schooling (OR 0.23, 95% CI 0.09-0.54) had a lower odds of text messaging. Having no earning member in the household was also associated with a lower odds of text messaging among males (OR 0.04, 95% CI 0.004-0.43). Males who lived in houses made of semiconcrete had higher odds of text messaging (OR 3.94, 95% CI 1.28-12.09).

Individual education (*P*=.04), household education (*P*<.001), satellite television service (*P*=.03), and smoking (*P*=.01) remained significantly associated with text messaging. Females who had no schooling (OR 0.24, 95% CI 0.06-0.97) or incomplete schooling (OR 0.23, 95% CI 0.12-0.46) had a lower odds of text messaging. Females who owned a television with satellite service had higher odds of text messaging (OR 2.06, 95% CI 1.08-3.93). Smoking behavior was associated with lower odds of text messaging (OR 0.45, 95% CI 0.26, 0.79).

**Table 3:** Multivariable analysis showing predictors of text messaging within the male and female study participants (N=904).

Variables		Text messaging			
		Males (n=305)		Females (n=599)	
		OR (95% CI)	*P* value	OR (95% CI)	*P* value
**Age (years)**					
	18-30	—^a^	—	1.81 (0.86-3.81)	.12
	31-40	—	—	1.95 (0.92-4.16)	.08
	41-50	—	—	2.18 (0.92-5.15)	.08
	50+^b^	—	—	—	—
**Education**					
	No school	—	—	0.24 (0.06-0.97)	.04
	Incomplete school	—	—	0.57 (0.15-2.24)	.42
	High school diploma	—	—	1.37 (0.26-7.12)	.71
	Some College or college graduate^b^	—	—	—	—
**Household education**					
	No school	0.05 (0.02-0.18)	<.001	0.10 (0.04-0.25)	<.001
	Incomplete school	0.23 (0.09-0.54)	.001	0.23 (0.12-0.46)	<.001
	High school diploma	0.47 (0.17-1.28)	.14	0.47 (0.21-1.02)	.06
	Some College or college graduate^b^	—	—	—	—
**Type of family**					
	Broken^b^	—	—	—	—
	Extended	—	—	0.57 (0.07-4.48)	.59
	Joint	—	—	1.81 (0.30-10.89)	.52
	Nuclear	—	—	0.80 (0.13-4.82)	.81
**Total earning members in the household**					
	No earning member	0.04 (0.004-0.43)	.01	—	—
	1 earning member	0.47 (0.19-1.16)	.10	—	—
	2 earning members	0.77(0.29-2.03)	.59	—	—
	3 or more earning members^b^	—	—	—	—
**Housing type**					
	Nonconcrete^b^	—	—	—	—
	Concrete	2.99 (0.99-9.03)	.05	1.66 (0.81-3.43)	.17
	Semiconcrete	3.94 (1.28-12.09)	.02	0.50 (0.24-1.06)	.07
**Type of toilet facility**					
	In-house^b^	—	—	—	—
	Public place	0.90 (0.48-1.68)	.74	1.14 (0.73-1.79)	.56
	Open defecation	0.42 (0.15-1.23)	.11	0.79 (0.39-1.62)	.53
**Television ownership**					
	No^b^	—	—	—	—
	Yes	1.81 (0.52-6.34)	.35	1.29 (0.59-2.82)	.52
**Television ownership with satellite television service**					
	No^b^	—	—	—	—
	Yes	2.01 (0.66-6.11)	.22	2.06 (1.08-3.93)	.03
**Smoking**					
	No^b^	—	—	—	—
	Yes	—	—	0.45 (0.26-0.79)	.01

^a^Empty cells indicate that the variables were not significant in the bivariate analysis for the respective gender group.

^b^Reference group.

## Discussion

### Principal Findings

The results of this study showed that more than half of the study participants owned a mobile phone (602/904, 66.3%), 49.3% (446/904) of them were familiar with sending text messages, and 24.3% (220/904) of them had internet service on their mobile phones. Variables associated with mobile phone ownership included gender, age, household educational level, number of earning members, housing type, and type of toilet facility. Variables associated with text messaging included the age of the study participant, individual education, household education, number of earning members in the household, housing type, and satellite television service. Variables significantly associated with internet access included gender, age, and household education. High-risk behaviors, including smoking and alcohol consumption were not significantly associated with mobile phone ownership, internet access, or text messaging.

This study identified important differences in the demographic and behavioral correlates of mobile phone ownership, access to the internet, and text messaging between males and females. Females were half as likely to own mobile phones compared with males (OR 0.53, 95% CI 0.37-0.76), less likely to have internet access (OR 0.79, 95% CI 0.56-1.11), or knew how to send text messages (OR 0.93, 95% CI 0.66-1.31). This finding is consistent with several similar studies in the literature [12]. Prior studies have shown that women in low- and middle-income countries (LMICs) are 14% less likely to own mobile phones compared with men. In addition, internet access is 12% lower among women compared with men [13].

Variables associated with mobile phone ownership between males and females included age, individual education, and housing type, in the adjusted analysis (*P*<.05). Being older than 30 years of age (OR 2.19, 95% CI 1.35-3.58), having no education (OR 0.31, 95% CI 0.14-0.66), and living in houses made of concrete (OR 2.49, 95% CI 1.50-4.14) were associated with mobile phone ownership. Some of these findings were not consistent with prior literature [3]. A prior study assessing determinants of household phone ownership in rural Bangladesh showed higher odds of mobile phone ownership among younger participants between the ages of 20 and 24 (OR 1.22, 95% CI 1.03-1.44), with lesser odds among participants aged 30 years or more (OR 0.95, 95% CI 0.77-1.18) [3]. A consistent finding of the highlighted literature with this study was the significance of wealth index measures (such as living in houses made of concrete) as a predictor of mobile phone ownership (*P*<.001). In particular, the highest quartile of wealth index was a key predictor of mobile phone ownership in rural Bangladesh [3].

In addition to age and individual education, the number of earning members in a household and housing type were predictors of text messaging between males and females in the adjusted analysis. Having 1 earning member or less was associated with reduced odds of text messaging (OR 0.547, 95% CI 0.329-0.909). This finding is consistent with prior literature in similar settings [14]. Such findings indicate that a high financial debt (especially in the absence of male family members), coupled with family responsibilities that affect finances (such as having several dependent family members), are possible mechanisms that explain the lower technology utilization among females [14].

Individual education was the sole predictor of internet access between males and females in the adjusted analysis. Having less than a high school education was significantly associated with reduced odds of internet access between males and females (OR 0.34, 95% CI 0.15-0.75). This finding was consistent with prior studies highlighting the role of literacy in internet utilization across LMICs. According to a qualitative analysis of socioeconomic correlates of the gender digital divide in Rwanda, a male participant stated as follows [15]:

For most Rwandan women, particular barriers are illiteracy, lack of familiarity with the main languages of computer technology and the internet, lack of operational training in computers, heavy household tasks, and lack of self-confidence

male, 50 years

The relevance of individual education as a key predictor of all three measures of technology used between males and females in this study is consistent with prior literature [12,14]. These findings have been attributed to patriarchal norms, which are arguably more prevalent in the South Asian context [12]. One of such patriarchal norms is the dedication of funds for educating male children, but rather preparing for the wedding of female children [14]. Such norms largely promote mobile phone ownership and technology usage among male family members, although limiting their use among women, who would often require permission to use mobile phones [16].

Our study findings comparing the differences in technology outcomes between gender groups highlighted disparate predictors of mobile phone ownership, internet access, and text messaging between males and females. For instance, individual education was a predominant factor associated with all three technology outcomes. Age and housing type were only predictive of mobile phone ownership and text messaging, and the number of earning members was only predictive of text messaging. These disparate findings across these predictors suggested that individual differences within gender groups may also be evident across the technology outcomes.

Subsequent investigations indicated that predictors of the technology outcomes differed significantly within males and females. These findings are suggestive of disparate levels of social class that are predominant even with slum settings, consistent with prior literature [17]. For instance, slum studies conducted in the Indian states of Chennai showed that individuals residing on the outskirts or margins of slums that adjoin wealthy neighborhoods are likely to absorb the culture of these wealthy Chennai neighborhoods. Owing to this, they were found to have higher levels of technology awareness and utilization [17]. It is also likely that such slum residences that are situated closer to wealthy neighborhoods may have lower crime and poverty rates, which are major predictors of the disparate access to technology by gender [16]. These findings can be extrapolated to this study in explaining the individual differences within gender categories with respect to the technology outcomes studied, as discussed below.

Our results showed that the type of toilet facility in households was significantly associated with mobile phone ownership. However, this association was different among males and females. Using a public toilet facility was not significantly associated with mobile phone ownership among males but was significant for females. In addition, open defecation was significantly associated with mobile phone ownership among females and males. These findings can be explained by prior literature indicating that open defecation is a marker of reduced SES [18]. In comparison to public places where slum residents have to pay around US $0.03 to US $0.04 to use a community public toilet, individuals practicing open defecation do not have to pay for this practice. The finding of open defecation being protective among females could be attributed to a higher sense of insecurity among females with using shared public latrines [19]. In addition, prior literature has indicated that although policies have been enacted to foster the provision of gender-specific toilets and infrastructure, females remain disproportionately affected by a lack of female-specific sanitation facilities compared with their male counterparts. In particular, findings have recorded a 66% disparity between toilets for men and women as of April 2019 [19]. Taken together, open defecation as a marker of reduced socio-economic status ultimately impacts female ownership of household assets and technologies [19].

Our study also showed that among females, household education was a predictor of all the technology outcomes, in the adjusted analysis. The finding that household education (and not individual education) was a key predictor of all the technology outcomes within females is consistent with the dominance of patriarchal norms within slum settings, which serve to marginalize women. In particular, gender inequities have been attributed to norms such as lack of prioritization of women’s education from the childhood stage, women being forced to get married before they can attain financial independence, family responsibilities preventing women from working outside their homes, or in-laws forcing women to work in low-profit family businesses rather than letting them earn independently, and much more [14]. These combined factors serve to increase women’s financial dependence on the men within their households; thus, the educational level of these women become less significant, as that of the household earning member takes precedence [14].

This in turn lends support to our study finding that having 1 earning member or less was a key predictor of both internet access and text messaging (corresponding to higher levels of technology usage) among men. Our study findings showed that among males, the number of earning members was a predictor of both mobile phone ownership and text messaging. This situation may be explained by the likelihood for women to depend more on the men in the households for financial sustenance; hence, the lack of a household earning member may significantly impair the financial status of the households. This phenomenon may then translate into a reduced ownership of household infrastructure and assets such as housing type (concrete vs semiconcrete vs nonconcrete) and television ownership, which are essentially markers of wealth, with higher values indicating better SES.

### Strengths and Limitations

A limitation of this study is the convenience approach employed in the identification of slums and, ultimately, the households that were interviewed. This approach may have introduced selection bias and affected the generalizability of the results to the entire population across Indian slums. This study is, however, generalizable to individuals residing in the unnotified slums included in this study. This study had several strengths. Our study findings provide an in-depth exploration of individual gender differences in the digital divide and highlight relevant measures of these differences. Although our study does not demonstrate any causal or quasi-causal claims, it highlights the possible areas of intervention that are in line with the identified predictors.

### Conclusions

Our study findings show disparate access to the technology outcomes *within* males and females in slum settings and lend support for further research to examine the causal mechanisms promoting these differences. Such mechanisms may proffer significant solutions to address the technology divide within gender groups and ultimately between gender groups. Specifically, our study findings suggest that improving household education is crucial to address the disparate access and utilization of mobile phones, internet, and text messaging among women in slum settings, owing to the consistency in household educational level as a predictor across all these technology indicators. In addition, the mechanisms by which the number of household earning members influences the disparate access to technology among men, call for further exploration. Finally, although our chosen study focus was on gender disparities in access to specific technology outcomes, future studies could explore the impact of the gender digital divide on access to health care and other health-related behaviors beyond those covered in this study. Internet and mobile phone usage are required for electronic health and mobile health technologies to promote the utilization of health care services.

## Appendix

Multimedia Appendix 1 Variables associated with mobile phone ownership, internet access, and text messaging among the study participants.

Multimedia Appendix 2 Predictors of mobile phone ownership, internet access, and text messaging among the study participants.

Multimedia Appendix 3 Differences in mobile phone ownership across gender categories.

Multimedia Appendix 4 Differences in internet access across gender categories.

Multimedia Appendix 5 Differences in text messaging across gender categories.

Multimedia Appendix 6 Predictors of mobile phone ownership, internet access, and text messaging between males and females.

Multimedia Appendix 7 Within-gender variation related to mobile phone ownership.

Multimedia Appendix 8 Within-gender variation related to internet access.

Multimedia Appendix 9 Text messaging within the male and female study participants.

## Abbreviations

ICT: Information and Communication Technology
IRB: Institutional Review Board
ITU: International Telecommunication Union
LMICs: low- and middle-income countries
OR: odds ratio
SES: socioeconomic status
UN: United Nations
