# Supplementary material for: Gender and the Digital Divide Across Urban Slums of New Delhi, India: Cross-Sectional Study
Source: J Med Internet Res. 2020 Jun 22;22(6):e14714. doi: 10.2196/14714 (PMC7338923; doi:10.2196/14714)
Supplement: Multimedia Appendix 1 [file jmir_v22i6e14714_app1.docx]

**Multimedia Appendix 1.** Variables associated with mobile phone ownership, internet access, and text messaging among the study participants (N=904).

| Characteristic | | Mobile phone (n=602) | *P* value | Internet access (n=220) | *P* value | Text messaging (n=446) | *P* value |
| --- | --- | --- | --- | --- | --- | --- | --- |
| Age (years), mean (SD) | | 35 (12) | <.001 | 34 (13) | <.001 | 35 (12) | <.001 |
| **Age (years), n (%)** | | | | | | | |
|  | 18-30 | 264 (43.8) | .05 | 113 (51.4) | .02 | 208 (46.7) | .02 |
|  | 31-40 | 177 (29.4) | .05 | 53 (24) | .02 | 119 (26.7) | .02 |
|  | 41-50 | 94 (16) | .05 | 31 (14) | .02 | 73 (16) | .02 |
|  | 50+ | 67 (11) | .05 | 23 (10) | .02 | 46 (10) | .02 |

| **Gender, n (%)** |
| --- |

|  | Male | 237 (39.4) | <.001 | 93 (42) | .002 | 170 (38.1) | <.001 |
| --- | --- | --- | --- | --- | --- | --- | --- |
|  | Female | 365 (61.7) | <.001 | 127 (57.7) | .002 | 276 (61.8) | <.001 |

| **Education, n (%)** |
| --- |

|  | No school | 189 (31.4) | <.001 | 48 (22) | <.001 | 104 (23.3) | <.001 |
| --- | --- | --- | --- | --- | --- | --- | --- |
|  | Incomplete school | 327 (54.3) | <.001 | 116 (52.7) | <.001 | 252 (56.5) | <.001 |
|  | High school diploma | 47 (8) | <.001 | 24 (11) | <.001 | 45 (10) | <.001 |
|  | Some college or college graduate | 39 (6) | <.001 | 32 (15) | <.001 | 42 (9) | <.001 |

| **Household education, n (%)** |
| --- |

|  | No school | 52 (9) | <.001 | 11 (5) | <.001 | 19 (4) | <.001 |
| --- | --- | --- | --- | --- | --- | --- | --- |
|  | Incomplete school | 312 (51.8) | <.001 | 72 (33) | <.001 | 206 (46.2) | <.001 |
|  | High school diploma | 114 (18.9) | <.001 | 52 (24) | <.001 | 98 (22) | <.001 |
|  | Some college or college graduate | 124 (20.6) | <.001 | 85 (39) | <.001 | 123 (27.5) | <.001 |

| **Type of family, n (%)** |
| --- |

|  | Broken | 8 (1) | .02 | 2 (1) | <.001 | 5 (1) | <.001 |
| --- | --- | --- | --- | --- | --- | --- | --- |
|  | Extended | 15 (2) | .02 | 7 (3) | <.001 | 13 (3) | <.001 |
|  | Joint | 193 (32.1) | .02 | 95 (43) | <.001 | 164 (36.8) | <.001 |
|  | Nuclear | 386 (64.1) | .02 | 116 (52.7) | <.001 | 264 (59.2) | <.001 |

| **Total earning members in the household, n (%)** |
| --- |

|  | No earning member | 6 (1) | .04 | 2 (1) | <.001 | 4 (1) | <.001 |
| --- | --- | --- | --- | --- | --- | --- | --- |
| member | 1 earning member | 351 (58.3) | .04 | 110 (50.0) | <.001 | 245 (54.9) | <.001 |
|  | 2 earning members | 176 (29.2) | .04 | 74 (34) | <.001 | 138 (30.9) | <.001 |
|  | 3 or more earning members | 65 (11) | .04 | 32 (15) | <.001 | 57 (13) | <.001 |

| **Housing type, n (%)** |
| --- |

|  | Nonconcrete | 43 (7) | <.001 | 14 (6) | .02 | 25 (6) | .03 |
| --- | --- | --- | --- | --- | --- | --- | --- |
|  | Concrete | 372 (61.7) | <.001 | 153 (69.5) | .02 | 299 (67.0) | .03 |
|  | Semi concrete | 186 (30.8) | <.001 | 52 (24) | .02 | 120 (27) | .03 |

| **Type of toilet facility, n (%)** |
| --- |

|  | In-house | 266 (44.2) | <.001 | 111 (50.5) | <.001 | 212 (47.5) | <.001 |
| --- | --- | --- | --- | --- | --- | --- | --- |
|  | Public place | 296 (49.2) | <.001 | 91 (41) | <.001 | 205 (45.9) | <.001 |
|  | Open defecation | 40 (7) | <.001 | 18 (8) | <.001 | 29 (7) | <.001 |

| **Television ownership, n (%)** |
| --- |

|  | No | 87 (14) | <.001 | 24 (11) | <.001 | 44 (10) | <.001 |
| --- | --- | --- | --- | --- | --- | --- | --- |
|  | Yes | 515 (85.6) | <.001 | 196 (89.1) | <.001 | 402 (90.1) | <.001 |

| **Television ownership with satellite television service, n (%)** |
| --- |

|  | No | 137 (22.8) | <.001 | 40 (19) | <.001 | 78 (18) | <.001 |
| --- | --- | --- | --- | --- | --- | --- | --- |
|  | Yes | 437 (72.6) | <.001 | 173 (78.6) | <.001 | 347 (77.8) | <.001 |

| **Internet usage (mobile phone), n (%)** |
| --- |

|  | No | 417 (69.2) | <.001 | 35 (16) | <.001 | 71 (16) | <.001 |
| --- | --- | --- | --- | --- | --- | --- | --- |
|  | Yes | 185 (30.7) | <.001 | 185 (84.1) | <.001 | 375 (84.1) | <.001 |

| **Texting (mobile phone), n (%)** |
| --- |

|  | No | 227 (37.7) | <.001 | 15 (7) | <.001 | 241 (54.0) | <.001 |
| --- | --- | --- | --- | --- | --- | --- | --- |
|  | Yes | 375 (62.3) | <.001 | 205 (93.2) | <.001 | 205 (46) | <.001 |

| **Smoking, n (%)** |
| --- |

|  | No | 466 (77.4) | .56 | 168 (76.3) | .50 | 360 (80.7) | .05 |
| --- | --- | --- | --- | --- | --- | --- | --- |
|  | Yes | 136 (22.6) | .56 | 52 (24) | .50 | 86 (19) | .05 |

| **Alcohol consumption, n (%)** |
| --- |

|  | No | 524 (87.0) | .08 | 191 (86.7) | .40 | 393 (88.1) | .80 |
| --- | --- | --- | --- | --- | --- | --- | --- |
|  | Yes | 78 (13) | .08 | 29 (13) | .40 | 53 (12) | .80 |
