# Supplementary material for: Gender and the Digital Divide Across Urban Slums of New Delhi, India: Cross-Sectional Study
Source: J Med Internet Res. 2020 Jun 22;22(6):e14714. doi: 10.2196/14714 (PMC7338923; doi:10.2196/14714)
Supplement: Multimedia Appendix 2 [file jmir_v22i6e14714_app2.docx]

**Multimedia Appendix 2.** Predictors of mobile phone ownership, internet access, and text messaging among the study participants (N=904).

| Variables | Mobile phone ownership (n=602) | | Internet access (n=220) | | Text messaging (n=446) | |
| --- | --- | --- | --- | --- | --- | --- |
|  | OR (95% CI) | *P* value | OR (95% CI) | *P* value | OR (95% CI) | *P* value |
|  |  |  |  |  |  |  |
| **Gender** | | | | | | |

|  | Female | | 0.51 (0.35-0.75) | <.001 | 0.65 (0.44-0.96) | .03 | 0.88 (0.61-1.26) | .48 |
| --- | --- | --- | --- | --- | --- | --- | --- | --- |
|  | Male^a^ | | N/A |  | N/A |  | N/A |  |
| **Age (years)** | | | | | | | | |
|  | 18-30 | | 1.60 (0.95-2.69) | .08 | 2.19 (1.15-4.19) | .02 | 1.76 (1.02-3.06) | .04 |
|  | 31-40 | | 1.96 (1.14-3.38) | .02 | 1.74 (0.89-3.41) | .11 | 1.51 (0.86-2.65) | .16 |
|  | 41-50 | | 1.99 (1.07-3.69) | .03 | 1.64 (0.79-3.36) | .18 | 2.12 (1.13-3.97) | .02 |
|  | 50+^a^ | | N/A | ⸺ | N/A | ⸺ | N/A | ⸺ |
| **Education** | | | | | | | | |
|  | No school | 0.72 (0.26-1.94) | | .51 | 0.43 (0.17-1.06) | .07 | 0.23 (0.08-0.68) | .01 |
|  | Incomplete school | 1.45 (0.54-3.87) | | .46 | 0.87 (0.37-2.02) | .75 | 0.58 (0.19-1.66) | .31 |
|  | High school diploma | 1.23 (0.39-3.86) | | .73 | 0.72 (0.27-1.88) | .49 | 0.79 (0.24-2.67) | .71 |
|  | Some college or college graduate^a^ | N/A | | ⸺ | N/A | ⸺ | N/A | ⸺ |
| **Household education** | | | | | | | | |
|  | No school | 0.30 (0.15-0.62) | | <.001 | 0.16 (0.07-0.36) | <.001 | 0.12 (0.06-0.26) | <.001 |
|  | Incomplete school | 0.51 (0.28-0.94) | | .03 | 0.16 (0.09-0.28) | <.001 | 0.26 (0.14-0.45) | <.001 |
|  | High school diploma | 0.57 (0.28-1.12) | | .10 | 0.49 (0.28-0.87) | .01 | 0.49 (0.26-0.95) | .03 |
|  | Some  College or college graduate^a^ | N/A | | ⸺ | N/A | ⸺ | N/A | ⸺ |
| **Type of family** | | | | | | | | |
|  | Extended | | 0.54 (0.11-2.59) | .44 | 0.56 (0.09-3.69) | .55 | 0.48 (0.09-2.49) | .38 |
|  | Joint | | 1.14 (0.30-4.29) | .85 | 0.97 (0.19-4.96) | .97 | 0.99 (0.25-3.98) | .99 |
|  | Nuclear | | 1.07 (0.29-3.96) | .91 | 0.48 (0.09-2.41) | .37 | 0.62 (0.16-2.42) | .49 |
|  | Broken^a^ | | N/A |  | N/A |  | N/A |  |

| **Total earning members in the household** | | | | | | | |
| --- | --- | --- | --- | --- | --- | --- | --- |
|  | No earning member | 0.27 (0.08-0.91) | .03 | 0.29 (0.06-1.59) | .16 | 0.22 (0.05-0.93) | .04 |
|  | 1 earning member | 1.09 (0.61-1.97) | .76 | 0.80 (0.44-1.46) | .47 | 0.85 (0.48-1.51) | .57 |
|  | 2 earning members | 1.31 (0.72-2.38) | .38 | 1.09 (0.59-1.99) | .78 | 1.06 (0.59-1.91) | .85 |
|  | 3 or more earning members^a^ | N/A | ⸺ | N/A | ⸺ | N/A | ⸺ |

| **Housing type** | | | | | | | | | |
| --- | --- | --- | --- | --- | --- | --- | --- | --- | --- |
|  | Concrete | | | 1.99 (1.13-3.49) | .02 | 1.18 (0.59-2.37) | .65 | 2.05 (1.13-3.72) | .02 |
|  | Semiconcrete | | | 1.13 (0.65-1.98) | .66 | 0.69 (0.34-1.43) | .32 | 1.02 (0.56-1.86) | .96 |
|  | Nonconcrete^a^ | | | N/A |  | N/A |  | N/A |  |
| **Type of toilet facility** | | | | | | | | | |
|  | Public place | | 1.56 (1.07-2.25) | | .02 | 0.81 (0.55-1.20) | .29 | 1.12 (0.78-1.60) | .55 |
|  | Open defecation | | 0.49 (0.29-0.84) | | .01 | 1.16 (0.59-2.26) | .66 | 0.76 (0.43-1.34) | .34 |
|  | In-house^a^ | | N/A | |  | N/A |  | N/A |  |
| **Television ownership** | | | | | | | | | |
|  | Yes | 1.22 (0.67-2.19) | | | .51 | 1.31 (0.60-2.87) | .49 | 1.59 (0.84-3.04) | .15 |
|  | No^a^ | N/A | | |  | N/A |  | N/A |  |
| **Television ownership with satellite television service** | | | | | | | | | |
|  | Yes | 1.63 (0.96-2.75) | | | .07 | 1.58 (0.83,2.98) | .17 | 1.94 (1.12-3.33) | .02 |
|  | No^a^ | N/A | | |  | N/A |  | N/A |  |

^a^Reference group (N/A).
