# Supplementary material for: Gender and the Digital Divide Across Urban Slums of New Delhi, India: Cross-Sectional Study
Source: J Med Internet Res. 2020 Jun 22;22(6):e14714. doi: 10.2196/14714 (PMC7338923; doi:10.2196/14714)
Supplement: Multimedia Appendix 3 [file jmir_v22i6e14714_app3.docx]

**Multimedia Appendix 3**. Differences in mobile phone ownership across gender categories (N=904)

|  | Mobile phone ownership (n=602) | | |
| --- | --- | --- | --- |
|  | Male | Female | *P* value |
|  | n=237 | n=365 |  |
|  |  |  |  |
| **Age (years), n (%)** |  |  | .002 |
| 18-30 | 91(47) | 167(45.7) |  |
| 31-40 | 57(24) | 120(32.9) |  |
| 41-50 | 46(19) | 48(13) |  |
| 50+ | 37(16) | 30(8) |  |
|  |  |  |  |
| **Education, n (%)** |  |  | <.001 |
| No school | 37(16) | 152(41.6) |  |
| Incomplete school | 154(64.9) | 173(47.4) |  |
| High school diploma | 26(11) | 21(6) |  |
| Some college/college graduate | 20(8) | 19(5) |  |
|  |  |  |  |
| **Household education, n (%)** |  |  | .74 |
| No school | 17(7) | 37(10) |  |
| Incomplete school | 127(53.6) | 185(50.7) |  |
| High school diploma | 44(19) | 70(19) |  |
| Some college/college graduate | 49(21) | 75(21) |  |
|  |  |  |  |
| **Type of family, n (%)** |  |  | .97 |
| Broken | 3(1) | 5(1) |  |
| Extended | 5(2) | 10(3) |  |
| Joint | 77(32) | 116(32) |  |
| Nuclear | 152(64.1) | 234(64.1) |  |
|  |  |  |  |
| **Total earning members in the household, n (%)** |  |  | .19 |
| No earning member | 2(1) | 4(1) |  |
| One earning member | 129(54.4) | 222(60.8) |  |
| Two earning members | 70(30) | 109(26) |  |
| Three or more earning members | 33(14) | 32(9) |  |
|  |  |  |  |
| **Housing type, n (%)** |  |  | .02 |
| Non-concrete | 12(5) | 31(9) |  |
| Concrete | 137(57.8) | 235(64.9) |  |
| Semi-concrete | 87(37) | 99(27) |  |
|  |  |  |  |
| **Type of toilet facility, n (%)** |  |  | .91 |
| In-house | 103(43.5) | 163(44.7) |  |
| Public place | 119(50.2) | 177(48.5) |  |
| Open defecation | 15(6) | 25(7) |  |
|  |  |  |  |
| **Television ownership, n (%)** |  |  | .29 |
| No | 32(14) | 55(15) |  |
| Yes | 205(86.5) | 310(84.9) |  |
|  |  |  |  |
| **Television ownership with satellite TV service*, n (%)** |  |  | .07 |
| No | 45(20) | 92(26) |  |
| Yes | 181(76.4) | 256(70.1) |  |
|  |  |  |  |
| **High-risk behaviors, n (%)** |  |  |  |
| Smoking |  |  | .001 |
| No | 164(69.2) | 302(82.7) |  |
| Yes | 73(31) | 63(17) |  |
|  |  |  |  |
| **Alcohol consumption, n (%)** |  |  | .02 |
| No | 197(83.1) | 327(89.6) |  |
| Yes | 40(17) | 38(10) |  |
