# Supplementary material for: Gender and the Digital Divide Across Urban Slums of New Delhi, India: Cross-Sectional Study
Source: J Med Internet Res. 2020 Jun 22;22(6):e14714. doi: 10.2196/14714 (PMC7338923; doi:10.2196/14714)
Supplement: Multimedia Appendix 4 [file jmir_v22i6e14714_app4.docx]

**Multimedia Appendix 4.** Differences in Internet access across gender categories (N=904)

|  | Internet access (n=220) | | |
| --- | --- | --- | --- |
|  | Male | Female | *P* value |
|  | n=93 | n=127 |  |
|  |  |  |  |
| **Age (years), n (%)** |  |  | .09 |
| 18-30 | 53(57) | 60(47) |  |
| 31-40 | 17(18) | 36(28) |  |
| 41-50 | 10(11) | 21(17) |  |
| 50+ | 13(14) | 10(8) |  |
|  |  |  |  |
| **Education, n (%)** |  |  | <.001 |
| No school | 9(10) | 39(312) |  |
| Incomplete school | 48(52) | 68(54) |  |
| High school diploma | 15(16) | 9(7) |  |
| Some college/college graduate | 21(23) | 11(9) |  |
|  |  |  |  |
| **Household education, n (%)** |  |  | .25 |
| No school | 4(1) | 7(6) |  |
| Incomplete school | 33(35) | 39(31) |  |
| High school diploma | 16(17) | 36(28) |  |
| Some college/college graduate | 40(43) | 45(35) |  |
|  |  |  |  |
| **Type of family, n (%)** |  |  | .78 |
| Broken | 1(1) | 1(1) |  |
| Extended | 2(2) | 5(4) |  |
| Joint | 38(41) | 57(45) |  |
| Nuclear | 52(56) | 64(50) |  |
|  |  |  |  |
| **Total earning members in the household, n (%)** |  |  | .14 |
| No earning member | 1(1) | 1(1) |  |
| One earning member | 38(41) | 72(57) |  |
| Two earning members | 36(39) | 38(30) |  |
| Three or more earning members | 17(18) | 15(12) |  |
|  |  |  |  |
| **Housing type, n (%)** |  |  | .01 |
| Non-concrete | 3(3) | 11(9) |  |
| Concrete | 59(64) | 94(74) |  |
| Semi-concrete | 30(33) | 22(17) |  |
|  |  |  |  |
| **Type of toilet facility, n (%)** |  |  | .73 |
| In-house | 44(47) | 67(53) |  |
| Public place | 41(44) | 50(39) |  |
| Open defecation | 8(9) | 10(8) |  |
|  |  |  |  |
| **Television ownership, n (%)** |  |  | .09 |
| No | 14(15) | 10(8) |  |
| Yes | 79(85) | 117(92.1) |  |
|  |  |  |  |
| **Television ownership with satellite TV service*, n (%)** |  |  | .42 |
| No | 19(21) | 21(17) |  |
| Yes | 70(79) | 103(81.1) |  |
|  |  |  |  |
| **High-risk behaviors, n (%)** |  |  |  |
| Smoking |  |  | .05 |
| No | 65(70) | 103(81.1) |  |
| Yes | 28(30) | 24(19) |  |
|  |  |  |  |
| **Alcohol consumption, n (%)** |  |  | .92 |
| No | 81(87) | 110(86.7) |  |
| Yes | 12(13) | 17(13) |  |
