# Supplementary material for: Gender and the Digital Divide Across Urban Slums of New Delhi, India: Cross-Sectional Study
Source: J Med Internet Res. 2020 Jun 22;22(6):e14714. doi: 10.2196/14714 (PMC7338923; doi:10.2196/14714)
Supplement: Multimedia Appendix 5 [file jmir_v22i6e14714_app5.docx]

**Multimedia Appendix 5.** Differences in text messaging across gender categories (N=904).

|  | Text messaging (n=446) | | |
| --- | --- | --- | --- |
|  | Male | Female | *P* value |
|  | n=170 | n=276 |  |
|  |  |  |  |
| **Age (years), n (%)** |  |  | .03 |
| 18-30 | 78(46) | 130(47.1) |  |
| 31-40 | 35(21) | 84(30) |  |
| 41-50 | 33(19) | 40(14) |  |
| 50+ | 24(14) | 22(8) |  |
|  |  |  |  |
| **Education, n (%)** |  |  | <.001 |
| No school | 15(9) | 92(33) |  |
| Incomplete school | 112(65.9) | 140(50.7) |  |
| High school diploma | 22(13) | 23(8) |  |
| Some college/college graduate | 21(12) | 21(8) |  |
|  |  |  |  |
| **Household education, n (%)** |  |  | 0.48 |
| No school | 5(3) | 14(5) |  |
| Incomplete school | 85(50) | 121(43.8) |  |
| High school diploma | 34(20) | 64(23) |  |
| Some college/college graduate | 46(27) | 77(28) |  |
|  |  |  |  |
| **Type of family, n (%)** |  |  | .73 |
| Broken | 2(1) | 3(1) |  |
| Extended | 4(2) | 9(3) |  |
| Joint | 58(34) | 106(38) |  |
| Nuclear | 106(62.3) | 158(57.2) |  |
|  |  |  |  |
| **Total earning members in the household, n (%)** |  |  | .03 |
| No earning member | 1(1) | 3(1) |  |
| One earning member | 83(49) | 162(58.7) |  |
| Two earning members | 54(32) | 84(31) |  |
| Three or more earning members | 31(18) | 26(9) |  |
|  |  |  |  |
| **Housing type, n (%)** |  |  | <.001 |
| Non-concrete | 6(4) | 19(7) |  |
| Concrete | 98(59) | 201(72.9) |  |
| Semi-concrete | 64(38) | 56(20) |  |
|  |  |  |  |
| **Type of toilet facility, n (%)** |  |  | .40 |
| In-house | 75(44) | 137(49.6) |  |
| Public place | 85(50) | 120(43.4) |  |
| Open defecation | 10(6) | 19(7) |  |
|  |  |  |  |
| **Television ownership, n (%)** |  |  | .94 |
| No | 17(10) | 27(10) |  |
| Yes | 153(90.0) | 249(90.2) |  |
|  |  |  |  |
| **Television ownership with satellite TV service*, n (%)** |  |  | .51 |
| No | 27(17) | 51(19) |  |
| Yes | 134(78.9) | 213(77.2) |  |
|  |  |  |  |
| **High-risk behaviors, n (%)** |  |  |  |
| **Smoking** |  |  | <.001 |
| No | 118(69.4) | 242(87.7) |  |
| Yes | 52(31) | 34(12) |  |
|  |  |  |  |
| **Alcohol consumption, n (%)** |  |  | .04 |
| No | 143(84.1) | 250(90.6) |  |
| Yes | 27(16) | 26(9) |  |
