# Supplementary material for: Gender and the Digital Divide Across Urban Slums of New Delhi, India: Cross-Sectional Study
Source: J Med Internet Res. 2020 Jun 22;22(6):e14714. doi: 10.2196/14714 (PMC7338923; doi:10.2196/14714)
Supplement: Multimedia Appendix 6 [file jmir_v22i6e14714_app6.docx]

**Multimedia Appendix 6.** Predictors of mobile phone ownership, internet access, and text messaging between males and females(N=904).

| Variables | | Mobile phone ownership (n=602) | | | Internet access (n=220) | | Text messaging (n=446) | |
| --- | --- | --- | --- | --- | --- | --- | --- | --- |
|  | | OR (95% CI) | | *P* value | OR (95% CI) | *P* value | OR (95% CI) | *P* value |
|  | |  | |  |  |  |  |  |
| **Gender** | | | | | | | | |
|  | Male^a^ | N/A | |  | N/A | N/A | N/A | N/A |
|  | Female | 0.53 (0.371-0.76) | | .001 | 0.79 (0.56-1.11) | 0.17 | 0.93 (0.66-1.31) | .68 |
| **Age (years)** | | | | | | | | |
|  | 18-30 | | 1.43 (0.90-2.24) | .13 | ⸺ | ⸺ | 1.31 (0.81-2.12) | .28 |
|  | 31-40 | | 2.19 (1.35-3.58) | .002 | ⸺ | ⸺ | 1.57 (0.95-2.59) | .08 |
|  | 41-50 | | 2.03 (1.17-3.52) | .01 | ⸺ | ⸺ | 2.26 (1.30-3.93) | .004 |
|  | 50+^a^ | | N/A |  | —– | ⸺ | N/A | ⸺ |
| **Education** | | | | | | | | |
|  | No school | 0.31 (0.14-0.66) | | .003 | 0.09 (0.04-0.17) | <.001 | 0.06 (0.03-0.15) | <.001 |
|  | Incomplete school | 0.92 (0.43-1.97) | | .82 | 0.21 (0.11-0.39) | <.001 | 0.23 (0.09-0.54) | .001 |
|  | High school diploma | 0.84 (0.326-2.16) | | .71 | 0.34 (0.15-0.75) | .01 | 0.43 (0.16-1.20) | .11 |
|  | Some college or college graduate^a^ | N/A | | N/A | N/A | N/A | N/A | N/A |
| **Total earning members in the** **household** | | | | | | | | |
|  | No earning member | ⸺ | | ⸺ | ⸺ | ⸺ | 0.16 (0.04-0.64) | .009 |
|  | 1 earning member | ⸺ | | ⸺ | ⸺ | ⸺ | 0.55 (0.33-0.91) | .02 |
|  | 2 earning members | ⸺ | | ⸺ | ⸺ | ⸺ | 0.86 (0.50-1.48) | .59 |
|  | 3 or more earning members^a^ | ⸺ | | ⸺ | ⸺ | ⸺ | ⸺ | ⸺ |
| **Housing type** | | | | | | | | |
|  | Nonconcrete^a^ | N/A | | N/A | N/A | N/A | N/A | N/A |
|  | Concrete | 2.49 (1.50-4.14) | | .0004 | 1.81 (0.97-3.39) | .06 | 2.69 (1.55-4.69) | .0004 |
|  | Semiconcrete | 1.08 (0.643-1.81) | | .77 | 0.79 (0.41-1.56) | .51 | 1.05 (0.59-1.86) | .87 |
| **Smoking** | | | | | | | | |
|  | No^a^ | N/A | | N/A | — | — | N/A | N/A |
|  | Yes | 0.96 (0.63-1.46) | | .85 | ⸺ | ⸺ | 0.71 (0.47-1.07) | .10 |
| **Alcohol consumption** | | | | | | | | |
|  | No^a^ | N/A | | N/A | — | — | N/A | N/A |
|  | Yes | 1.45 (0.832-2.51) | | .19 | ⸺ | ⸺ | 1.17 (0.69-1.97) | .55 |

^a^ Reference group (N/A).

^b^ Empty cells indicate that the variables were not significant in the bivariate analysis for the applicable technology category
