# Supplementary material for: Gender and the Digital Divide Across Urban Slums of New Delhi, India: Cross-Sectional Study
Source: J Med Internet Res. 2020 Jun 22;22(6):e14714. doi: 10.2196/14714 (PMC7338923; doi:10.2196/14714)
Supplement: Multimedia Appendix 7 [file jmir_v22i6e14714_app7.docx]

**Multimedia Appendix 7**. Within gender variation related to mobile phone ownership (N=904)

|  | **Male** | | | **Female** | | |
| --- | --- | --- | --- | --- | --- | --- |
|  | Yes  n=237 | No  n=68 | *P* value | Yes  n=365 | No  n=234 | *P*  value |
|  |  |  |  |  |  |  |
| **Age (years), n (%)** |  |  | .30 |  |  | <.001 |
| 18-30 | 97(41) | 28(41) |  | 167(45.8) | 106(45.2) |  |
| 31-40 | 57(24) | 16(24) |  | 120(32.9) | 53(23) |  |
| 41-50 | 46(19) | 8(12) |  | 48(13) | 30(13) |  |
| 50+ | 37(16) | 16(24) |  | 30(8) | 45(19) |  |
|  |  |  |  |  |  |  |
| **Education, n (%)** |  |  | <.001 |  |  | <.001 |
| No school | 37(16) | 33(49) |  | 152(41.6) | 154(65.8) |  |
| Incomplete school | 154(64.9) | 25(37) |  | 173(47.4) | 66(28) |  |
| High school diploma | 26(11) | 6(9) |  | 21(6) | 8(3) |  |
| Some college/college graduate | 20(8) | 4(6) |  | 19(5) | 6(3) |  |
|  |  |  |  |  |  |  |
| **Household education, n (%)** |  |  | <.001 |  |  | <.001 |
| No school | 17(7) | 19(28) |  | 356(10) | 71(30) |  |
| Incomplete school | 127(53.6) | 31(46) |  | 185(50.7) | 110(47.0) |  |
| High school diploma | 44(19) | 9(13) |  | 70(19) | 32(14) |  |
| Some college/college graduate | 49(21) | 9(13) |  | 75(21) | 21(9) |  |
|  |  |  |  |  |  |  |
| **Type of family, n (%)** |  |  | .31 |  |  | .05 |
| Broken | 3(1) | 1(1) |  | 5(1) | 7(3) |  |
| Extended | 5(2) | 2(3) |  | 10(3) | 16(7) |  |
| Joint | 77(32) | 14(21) |  | 116(32) | 70(30) |  |
| Nuclear | 152(64.1) | 51(75) |  | 234(64.1) | 141(60.3) |  |
|  |  |  |  |  |  |  |
| **Total earning members in the household*, n (%)** |  |  | <.001 |  |  | .52 |
| No earning member | 2(1) | 7(10) |  | 4(1) | 7(3) |  |
| One earning member | 129(54.4) | 37(54.4) |  | 222(60.8) | 146(62.3) |  |
| Two earning members | 70(30) | 15(22) |  | 106(29.0) | 57(25) |  |
| Three or more earning members | 33(14) | 9(13) |  | 32(9) | 22(9) |  |
|  |  |  |  |  |  |  |
| **Housing type*, n (%)** |  | <.001 | <.001 |  |  | <.001 |
| Non-concrete | 12(5) | 15(22) |  | 31(9) | 28(12) |  |
| Concrete | 137(57.8) | 19(28) |  | 235(64.4) | 105(44.9) |  |
| Semi-concrete | 87(37) | 33(49) |  | 99(27) | 101(43.2) |  |
|  |  |  |  |  |  |  |
| **Type of toilet facility, n (%)** |  | <.001 | <.001 |  |  | <.001 |
| In-house | 103(43.5) | 19(28) |  | 163(44.7) | 92(39) |  |
| Public place | 119(50.2) | 32(47) |  | 177(48.5) | 81(35) |  |
| Open defecation | 15(6) | 17(25) |  | 25(7) | 61(26) |  |
|  |  |  |  |  |  |  |
| **Television ownership, n (%)** |  |  | <.001 |  |  | <.001 |
| No | 32(14) | 35(51) |  | 55(15) | 77(33) |  |
| Yes | 205(86.4) | 33(19) |  | 310(84.9) | 157(67.1) |  |
|  |  |  |  |  |  |  |
| **Television ownership with satellite TV service*, n (%)** |  |  | <.001 |  |  | <.001 |
| No | 45(20) | 41(60) |  | 92(26) | 100(44) |  |
| Yes | 181(76.4) | 27(40) |  | 256(70.1) | 128(54.7) |  |
|  |  |  |  |  |  |  |
| **High-risk behaviors, n (%)** |  |  |  |  |  |  |
| Smoking |  |  | .02 |  |  | .24 |
| No | 164(69.2) | 37(54) |  | 302(82.7) | 202(86.3) |  |
| Yes | 73(31) | 31(46) |  | 63(17) | 32(14) |  |
|  |  |  |  |  |  |  |
| **Alcohol consumption, n (%)** |  |  | .89 |  |  | .14 |
| No | 197(83.1) | 57(84) |  | 327(89.6) | 218(93.1) |  |
| Yes | 40(17) | 11(16) |  | 38(10) | 16(7) |  |
