# Supplementary material for: Gender and the Digital Divide Across Urban Slums of New Delhi, India: Cross-Sectional Study
Source: J Med Internet Res. 2020 Jun 22;22(6):e14714. doi: 10.2196/14714 (PMC7338923; doi:10.2196/14714)
Supplement: Multimedia Appendix 8 [file jmir_v22i6e14714_app8.docx]

**Multimedia Appendix 8**. Within gender variation related to internet access (N=904)

|  | Male | | | Female | | |
| --- | --- | --- | --- | --- | --- | --- |
|  | Yes  n=93 | No  n=212 | *P*  value | Yes  n=127 | No  n=472 | *P*  value |
|  |  |  |  |  |  |  |
| **Age (years), n (%)** |  |  | .002 |  |  | .22 |
| 18-30 | 53(57) | 72(34) |  | 60(47) | 213(45.1) |  |
| 31-40 | 17(18) | 56(26) |  | 36(28) | 137(29.0) |  |
| 41-50 | 10(11) | 44(21) |  | 21(17) | 57(12) |  |
| 50+ | 13(14) | 40(19) |  | 10(8) | 65(14) |  |
|  |  |  |  |  |  |  |
| **Education, n (%)** |  |  | <.001 |  |  | <.001 |
| No school | 9(10) | 61(29) |  | 39(312) | 267(56.6) |  |
| Incomplete school | 48(52) | 131(61.8) |  | 68(54) | 171(36.2) |  |
| High school diploma | 15(16) | 17(8) |  | 9(7) | 20(4) |  |
| Some college/college graduate | 21(23) | 3(1) |  | 11(9) | 14(3) |  |
|  |  |  |  |  |  |  |
| **Household education, n (%)** |  |  | <.001 |  |  | <.001 |
| No school | 4(1) | 32(15) |  | 7(6) | 99(21) |  |
| Incomplete school | 33(35) | 125(58.9) |  | 39(31) | 256(54.2) |  |
| High school diploma | 16(17) | 37(17) |  | 36(28) | 66(14) |  |
| Some college/college graduate | 40(43) | 18(8) |  | 45(35) | 51(11) |  |
|  |  |  |  |  |  |  |
| **Type of family, n (%)** |  |  | .05 |  |  | .002 |
| Broken | 1(1) | 3(1) |  | 1(1) | 11(2) |  |
| Extended | 2(2) | 5(2) |  | 5(4) | 21(4) |  |
| Joint | 38(41) | 53(25) |  | 57(45) | 129(27.3) |  |
| Nuclear | 52(56) | 151(71.2) |  | 64(50) | 311(65.9) |  |
|  |  |  |  |  |  |  |
| **Total earning members in the household*, n (%)** |  |  | .003 |  |  | .33 |
| No earning member | 1(1) | 8(4) |  | 1(1) | 10(2) |  |
| One earning member | 38(41) | 128(60.4) |  | 72(57) | 296(62.7) |  |
| Two earning members | 36(39) | 49(23) |  | 38(30) | 125(26.5) |  |
| Three or more earning members | 17(18) | 25(12) |  | 15(12) | 39(8) |  |
|  |  |  |  |  |  |  |
| **Housing type*, n (%)** |  |  | .01 |  |  | <.001 |
| Non-concrete | 3(3) | 24(11) |  | 11(9) | 48(10) |  |
| Concrete | 59(64) | 97(46) |  | 94(74) | 246(52.1) |  |
| Semi-concrete | 30(33) | 90(43) |  | 22(17) | 178(37.7) |  |
|  |  |  |  |  |  |  |
| **Type of toilet facility, n (%)** |  |  | .22 |  |  | .01 |
| In-house | 44(47) | 78(37) |  | 67(53) | 188(39.8) |  |
| Public place | 41(44) | 110(51.9) |  | 50(39) | 208(44.1) |  |
| Open defecation | 8(9) | 24(11) |  | 10(8) | 76(16) |  |
|  |  |  |  |  |  |  |
| **Television ownership, n (%)** |  |  | .05 |  |  | <.001 |
| No | 14(15) | 53(25) |  | 10(8) | 122(26) |  |
| Yes | 79(85) | 159(75.0) |  | 117(92.1) | 350(74.2) |  |
|  |  |  |  |  |  |  |
| **Television ownership with satellite TV service*, n (%)** |  |  | .05 |  |  | <.001 |
| No | 19(21) | 67(33) |  | 21(17) | 171(36.2) |  |
| Yes | 70(79) | 138(65.1) |  | 103(81.1) | 281(59.5) |  |
|  |  |  |  |  |  |  |
| **High-risk behaviors, n (%)** |  |  |  |  |  |  |
| Smoking |  |  | .33 |  |  | .29 |
| No | 65(70) | 136(64.2) |  | 103(81.1) | 401(84.2) |  |
| Yes | 28(30) | 76(36) |  | 24(19) | 71(15) |  |
|  |  |  |  |  |  |  |
| **Alcohol consumption, n (%)** |  |  | .24 |  |  | .05 |
| No | 81(87) | 173(81.6) |  | 110(86.6) | 435(91.4) |  |
| Yes | 12(13) | 39(18) |  | 17(13) | 37(8) |  |
