# Supplementary material for: Gender and the Digital Divide Across Urban Slums of New Delhi, India: Cross-Sectional Study
Source: J Med Internet Res. 2020 Jun 22;22(6):e14714. doi: 10.2196/14714 (PMC7338923; doi:10.2196/14714)
Supplement: Multimedia Appendix 9 [file jmir_v22i6e14714_app9.docx]

**Multimedia Appendix 9.** Text messaging within the male and female study participants.

|  | Male | | | Female | | |
| --- | --- | --- | --- | --- | --- | --- |
|  | Yes  n=170 | No  n=135 | *P*  value | Yes  n=276 | No  n=323 | *P*  value |
|  |  |  |  |  |  |  |
| **Age (years), n (%)** |  |  | .07 |  |  | .02 |
| 18-30 | 78(46) | 47(35) |  | 130(47.1) | 143(44.3) |  |
| 31-40 | 35(21) | 38(28) |  | 84(30) | 89(28) |  |
| 41-50 | 33(19) | 21(16) |  | 40(14) | 38(12) |  |
| 50+ | 24(14) | 29(21) |  | 22(8) | 53(16) |  |
|  |  |  |  |  |  |  |
| **Education, n (%)** |  |  | <.001 |  |  | <.001 |
| No school | 15(9) | 55(41) |  | 92(33) | 214(66.3) |  |
| Incomplete school | 112(65.9) | 67(50) |  | 140(50.7) | 99(31) |  |
| High school diploma | 22(13) | 10(7) |  | 23(8) | 6(2) |  |
| Some college/college graduate | 21(12) | 3(2) |  | 21(8) | 4(1) |  |
|  |  |  |  |  |  |  |
| **Household education, n (%)** |  |  | <.001 |  |  | <.001 |
| No school | 5(3) | 31(23) |  | 14(5) | 92(28) |  |
| Incomplete school | 85(50) | 73(54) |  | 121(43.8) | 174(53.8) |  |
| High school diploma | 34(20) | 19(14) |  | 64(23) | 38(12) |  |
| Some college/college graduate | 46(27) | 12(9) |  | 77(28) | 19(6) |  |
|  |  |  |  |  |  |  |
| **Type of family, n (%)** |  |  | .33 |  |  | .002 |
| Broken | 2(1) | 2(1) |  | 3(1) | 9(3) |  |
| Extended | 4(2) | 3(2) |  | 9(3) | 17(5) |  |
| Joint | 58(34) | 33(24) |  | 106(38) | 80(25) |  |
| Nuclear | 106(62.3) | 97(72) |  | 158(57.2) | 217(67.2) |  |
|  |  |  |  |  |  |  |
| **Total earning members in the household*, n (%)** |  |  | .001 |  |  | .24 |
| No earning member | 1(1) | 8(6) |  | 3(1) | 8(2) |  |
| One earning member | 83(49) | 83(62) |  | 162(58.7) | 206(63.8) |  |
| Two earning members | 54(32) | 31(23) |  | 84(31) | 79(25) |  |
| Three or more earning members | 31(18) | 11(8) |  | 26(9) | 28(9) |  |
|  |  |  |  |  |  |  |
| **Housing type*, n (%)** |  |  | <.001 |  |  | <.001 |
| Non-concrete | 6(4) | 21(16) |  | 19(7) | 40(12) |  |
| Concrete | 98(59) | 58(43) |  | 201(72.8) | 139(43.0) |  |
| Semi-concrete | 64(38) | 56(41) |  | 56(20) | 144(44.6) |  |
|  |  |  |  |  |  |  |
| **Type of toilet facility, n (%)** |  |  | .01 |  |  | <.001 |
| In-house | 75(44) | 47(35) |  | 137(49.6) | 118(36.5) |  |
| Public place | 85(50) | 66(49) |  | 120(43.4) | 138(42.7) |  |
| Open defecation | 10(6) | 22(16) |  | 19(7) | 67(21) |  |
|  |  |  |  |  |  |  |
| **Television ownership, n (%)** |  |  | <.001 |  |  | <.001 |
| No | 17(10) | 50(37) |  | 27(10) | 105(32.5) |  |
| Yes | 153(90.0) | 85(63) |  | 249(90.2) | 218(67.5) |  |
|  |  |  |  |  |  |  |
| **Television ownership with satellite TV service*, n (%)** |  |  | <.001 |  |  | <.001 |
| No | 27(17) | 59(44) |  | 51(19) | 141(43.7) |  |
| Yes | 134(78.8) | 74(56) |  | 213(77.1) | 171(52.9) |  |
|  |  |  |  |  |  |  |
| **High-risk behaviors, n (%)** |  |  |  |  |  |  |
| Smoking |  |  | .15 |  |  | .03 |
| No | 118(69.4) | 83(61) |  | 242(87.7) | 262(81.1) |  |
| Yes | 52(31) | 52(39) |  | 34(12) | 61(19) |  |
|  |  |  |  |  |  |  |
| **Alcohol consumption, n (%)** |  |  | .66 |  |  | .75 |
| No | 143(84.1) | 111(82.2) |  | 250(90.5) | 295(91.3) |  |
| Yes | 27(16) | 24(18) |  | 26(9) | 28(9) |  |
